# Annual Meeting of the N. Y. State Medical Society

**Published:** 1850-03

**Authors:** 


*Annual Meeting of the N. Y. State Medical Society.*—The Annual meeting of the N. Y. State Medical Society was held at Albany on the 5th ult. About thirty members and delegates were present. A number of reports, and voluntary communications were received, and referred to the Committee on Publications. The volume of Transactions will probably be larger than usual. We are glad to learn that the Legislature have passed a resolution providing for the publication of the Transactions, etc., at the expense of the State. We reserve remarks on the papers communicated, until the published transactions appear, which will probably not be for two or three months to come.

The subject of Free Medical Schools occasioned some discussion. Some resolutions on this subject were submitted, and finally laid on the table. The entire forenoon of the last day of the session was occupied with the discussion of the identity of Typhus and Typhoid fevers. Prof. Clark, of the city of New York, opened the discussion, by appointment at the meeting of the Society in 1849. The substance of his remarks will appear in the Transactions.

Special committees were appointed to report, at the meeting of 1851, on Epidemic Cholera, and Fever, in addition to standing committees on several scientific subjects.

The Society continued in session from the 5th to the 7th inclusive. The meeting was spirited, interesting, and profitable, and we should have been glad to have seen a larger attendance. A large proportion of the counties in the State were not represented. It strikes us that it would conduce to greater usefulness of the Society, if its doors were thrown open to all regular members of county Societies throughout the State, restricting the right of voting, so that each Society, and Medical School, should have but a single vote in all important matters, exclusive of scientific subjects. Some plan of popularizing the Society with the profession, (if this expression be allowable) would, as it seems to us, not only increase the attend ance, but render its meetings more interesting and useful. It seems to us, also, worth considering, whether it might not be an improvement to meet annually at a different part of the State, selecting, in succession, the larger and more accessible towns, Utica, Syracuse, Rochester, Buffalo, etc. This would not only, in the long run, better accomodate the Profession throughout the State, but a local influence would be exerted in the place and vicinity of each meeting, which would be salutary. We throw out these suggestions to be taken for what they are worth.

The following are the officers of the Society elected for 1850:

Dr. Alexander H. Thompson, President.
Jenks S. Sprague, Vice President.
Thomas Hun, Secretary.
P. Van O'Linda, Treasurer.

Censors.—*Southern District.*—Drs. J. R. Manly, John Cheesman, Chas. S. J. Goodrich. *Eastern District.*—Drs. Joel A. Wing, Tho's W. Blatchford, T. R. Beck. *Middle District.*—Drs. Augustus Willard, S. H. French, John McCall. *Western District.*—Drs. Bryant Burwell, D. T. Jones, John Coates.

Committee on Correspondence.—Drs. Alonzo Clark, Thomas W. Blatchford, John H. Wheeler, Hiram Corliss, Helon F. Noyes, Augustus Willard, Phineas H. Burdick, Bryant Burwell.

Permanent Members Elected.—Drs. Alonzo Clark, Nathaniel Miller, —— Brinsmade, Hiram Corliss, Helon F. Noyes, S. H. French, Daniel T. Jones, Seth H. Pratt.

Committee of Publication.—Drs. T. R. Beck, James McNaughton, Joel A. Wing.

Committee on Prize Questions and Disertations.—Drs. James McNaughton, T. R. Beck, P. Van Buren, Joel A. Wing, Thomas Hun.

Delegates to National Medical Association.—Drs. Charles S. Goodrich, A. H. Stevens, J. R. Manly, J. R. Wood, J. C. Cheesman, Hiram Corliss, S. H. French, T. W. Blatchford, R. G. Frany, S. Snow, Henry Mitchell, Augustus Willard, J. McCall, J. S. Sprague, N. H. Dehning, B. Burwell, T. Spencer, A. Thompson, J. P. White, G. W. Bradford.

